# Duloxetine-induced rapid eye movement sleep behavior disorder: a case report

**DOI:** 10.1186/s12888-017-1535-4

**Published:** 2017-11-21

**Authors:** Lu Tan, Junying Zhou, Linghui Yang, Rong Ren, Ye Zhang, Taomei Li, Xiangdong Tang

**Affiliations:** Sleep Medicine Center, Laboratory of Anaesthesia & Critical Care Medicine, Translational Neuroscience Center, West China Hospital, Sichuan University, Chengdu, China

**Keywords:** Duloxetine, Rapid eye movement sleep behavior disorder, Polysomnography

## Abstract

**Background:**

Tricyclic antidepressants and selective serotonin reuptake inhibitors have been reported to induce the symptoms of rapid eye movement (REM) sleep behavior disorder (RBD) or to exacerbate REM sleep without atonia. With this case report, we found an association between typical RBD and duloxetine, a serotonin-noradrenaline reuptake inhibitor.

**Case presentation:**

We present a case of a 62-year-old woman who experienced enactment behaviors with violent dreams that were associated with increased tonic or phasic chin electromyography activity during REM sleep after treated with duloxetine. RBD symptoms were gradually reduced and completely ceased after discontinuation of duloxetine for 37 days.

**Conclusion:**

The current case appears to be the first observation of duloxetine-induced RBD. We describe features of RBD induced by duloxetine that are similar and different from that induced by tricyclic antidepressants and selective serotonin reuptake inhibitors.

## Background

Rapid eye movement (REM) sleep behavior disorder (RBD) is characterized by the loss of normal skeletal muscle atonia during REM sleep and the emergence of purposeful complex motor activity associated with vivid dreams [1, 2]. It has been widely known that some antidepressants such as tricyclic antidepressants (TCA) and selective serotonin reuptake inhibitors (SSRI) can induce RBD [3]. The prevalence of antidepressant-induced RBD varies across studies. In a group of 1235 outpatient psychiatric patients, the lifetime prevalence for RBD-like disorder was 5.8%. Among patients taking SSRIs, the prevalence of RBD-like disorder was 5% [4]. Another large-scale study reported that 12.2% of patients taking antidepressants had REM sleep without atonia (RSWA) but only 0.48% of the patients had RBD [5].

In addition to TCAs and SSRIs, serotonin-norepinephrine reuptake inhibitors (SNRI) such as venlafaxine and duloxetine are used clinically as antidepressants. However, there is little information on the potential association between SNRI use and RBD or RSWA [5–7]. McCarter et al. analyzed RSWA in adults receiving antidepressants, with and without RBD. They found that patients taking SNRIs had elevated RSWA, unfortunately, the association between duloxetine and RSWA was not reported in this work [7]. The current case appears to be the first observation of duloxetine-induced RBD.

## Case presentation

**Fig. 1:**
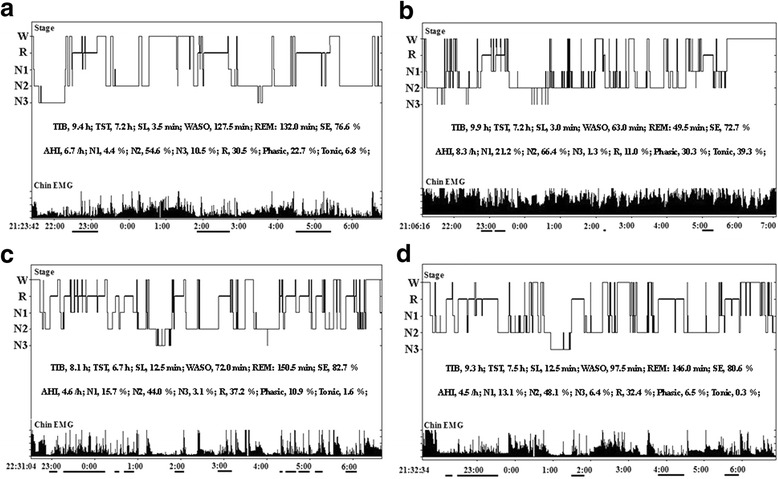
Changes in sleep histogram and chin electromyography tonus with treatment of duloxetine. **a** First PSG for the patient after duloxetine, 60 mg/day, for 7 months. **b** Second PSG for patient after duloxetine 60 mg/day, for 2.7 years. **c** Third PSG after discontinuation of duloxetine for 9 days. **d** Fourth PSG after discontinuation of duloxetine for 37 days. Lines under the x-axis indicate episodes of rapid eye movement sleep. PSG, polysomnography; W, wake; N1–3, non-rapid eye movement sleep 1–3; R, rapid eye movement sleep; TIB, time in bed; TST, total sleep time; SL, sleep latency; WASO: wake after sleep onset; REM: rapid eye movement; SE, sleep efficiency; AHI, apnea-hypopnea index; EMG, electromyography

A 62-year old woman presented at the psychiatry clinic with dizziness, back pain, gastrointestinal discomfort and anxiety. She was diagnosed with somatoform disorder and treated with duloxetine, 60 mg per day, for 7 months. Although the somatic symptoms and related mood disorders improved, she also complained about frequent awakening during night and was unsatisfied with her sleep quality. For this reason, she sought help from our sleep medicine center and an overnight polysomnography (PSG) study was arranged. The examination showed that she had a total sleep time (TST) of 7.2 h with apnea-hypopnea index (AHI) of 6.7 /h (AHI is a parameter used to classify the severity of sleep apnea, the cut-off point for mild, moderate and severe sleep apnea is 5 /h, 15 /h and 30 /h). Tonic and phasic electromyography (EMG) activity were scored manually according to the criteria of the 2007 American Academy of Sleep Medicine (AASM) manual [8]. During the 264 30-s epochs (132 min) of REM, we found that 29.5% of REM sleep had increased chin EMG activity with a 6.8% increase for tonic activity and a 22.7% for phasic activity (Fig. 1a). However, the patient did not complain of any abnormal behaviors during nocturnal sleep except for more vivid dreams. Moreover, we did not notice significant movements during REM sleep from the video of the evaluation night. As the symptoms of somatoform disorder had significantly improved, the treatment of duloxetine was continued for about 2 years.

During the following 2 years, the patient’s daughter noticed that she had occasional speaking and yelling during the night (a few times per month). The symptoms gradually worsened over time (two or three times per week), with not only frequent vocalization during sleep but also some enactment behaviors with violent dreams. For example, she once fell from bed during sleep when she had a vivid dream of running and chasing someone. In another instance, she dreamed of biting something, but she was actually biting the hand of her grandson. She did not have any co-medication or alcohol/substance use. Similarly, she had no medical histories of sleepwalking, daytime sleepiness, neurodegenerative diseases like Parkinson’s disease (PD) or any clinical symptoms of cognitive dysfunction. In addition, she had no family histories of RBD and neurodegenerative diseases. Neurological examinations were unremarkable. Brain magnetic resonance imaging (MRI) showed no clinically significant abnormalities. The scores of the Epworth Sleep Scale (ESS) and the RBD questionnaire (RBDQ-HK) were 0 and 60 (cut-off point of total score is 18), respectively. The score of the Hamilton Depression Scale (HAMD) was 7 but the score of Hamilton Anxiety Scale (HAMA) was increased mildly to 10. We then arranged an overnight PSG examination for her. We found that most of epochs of REM sleep had increased tonic chin EMG activity (39.3%) and phasic chin EMG activity (30.3%) (Fig. 1b). Moreover, the video monitoring revealed abnormal behavior episodes (waving hands or moving legs) during REM sleep.

**Fig. 2:**
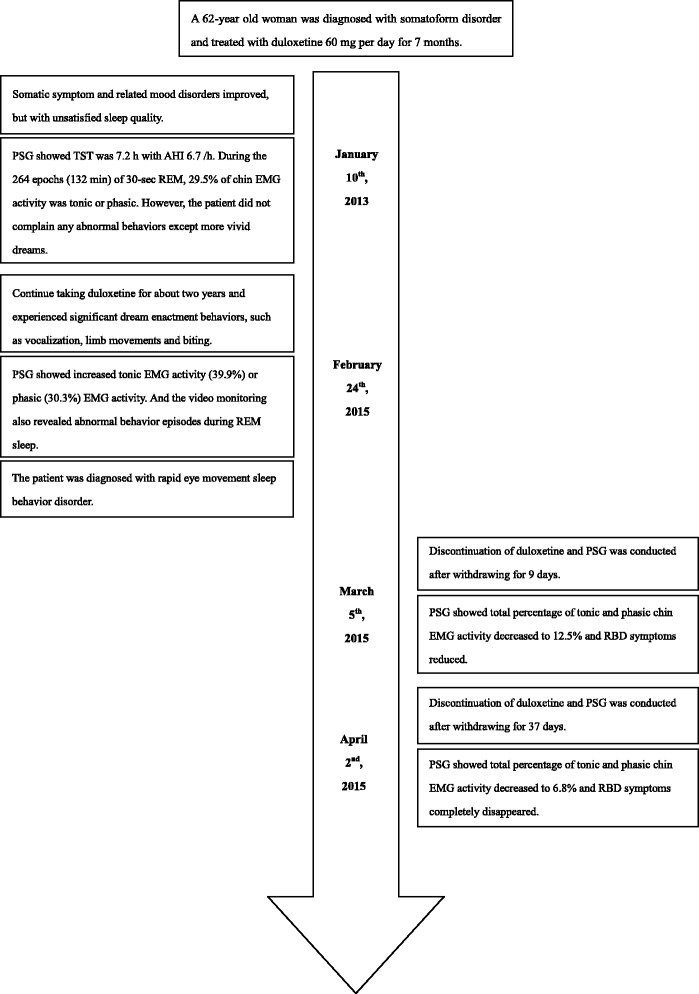
Timeline of clinical history and medication use. PSG, polysomnography; TST, total sleep time; AHI, apnea-hypopnea index; REM, rapid eye movement sleep; EMG, electromyography; RBD, rapid eye movement sleep behavior disorder

The clinical manifestation and video PSG suggested that the patients met the diagnostic criteria of RBD with dream-enactment behaviors during REM sleep and RSWA. In view of the temporal association between medication and RBD and a lack of evidence suggesting RBD secondary to other comorbidity, we recommended that the patient to discontinue the use of duloxetine. Two overnight video PSG sessions were conducted after discontinuing duloxetine for 9 days and 37 days. As shown in Fig. 1c and d, the total percentage of tonic and phasic chin EMG activity decreased to 12.5% and 6.8%, respectively. The most important change was that RBD symptoms gradually reduced with the discontinuation of duxoletine and completely disappeared after 37 days. The timeline of clinical history and medication history are summarized in Fig. 2.

## Discussion and conclusions

The RBD symptoms occurred during the period of duloxetine use and disappeared after drug discontinuation, which indicated that it was indeed a case of duloxetine-induced RBD. This finding suggests that SNRIs can induce RBD. We herein report some similar and different features of SNRI-induced RBD from those associated with TCAs or SSRIs.

Kluge et al. found that patients with major depression taking duloxetine for 7–14 days presented decreased amounts of REM sleep and increased REM sleep latency compared to pre-treatment [9]. We also found similar results in the current case in that REM sleep latency increased from 66 min to 100.5 min and the amount of REM sleep decreased from 132 min to 49.5 min after the patient took duloxetine for about 2 years. In addition, Zhang et al. found that increased tonic and phasic RSWA were correlated with prolonged REM latency [10] and Winkelman et al. found that tonic submental EMG activity was positively correlated with REM latency and inversely correlated with REM time [11]. In our case, we found that phasic EMG activity was positively correlated with REM latency (*r* = 0.952, *p* = 0.048) and that tonic EMG activity was negatively correlated with REM sleep duration (*r* = −0.998, *p* = 0.002). Moreover, significantly increased EMG activity was observed not only in REM sleep, but also in non-REM sleep (Fig. 1b). Similarly, other antidepressants such as imipramine and paroxetine have been reported as augmenting EMG activity during different sleep stages [12, 13]. Thus, EMG activity in antidepressant induced RBD may help differentiate it from typical RBD, which has not been reported to have increased EMG activity in non-REM sleep.

There were also two unique features in this case. First, the latency to drug induced RBD was longer than the interval, usually less than 6 months, previously reported for SSRI induced RBD [10, 14]. Actually, in our case, excessive EMG activity during REM sleep was found during the first PSG assessment taken when the patient had been taking duloxetine for 7 months, but without typical RBD symptoms. There may be two reasons to explain the long interval from beginning duloxetine to the emergence of typical RBD symptoms. One is that the pathway of RBD induced by duloxetine may be different from that by SSRI, and that duloxetine took longer to produce the cumulative effect progressing from increased EMG activity to clinical RBD symptoms. The other is that the patient might have had mild abnormal behaviors, such as vocalization related to REM sleep, in the early process of drug induced RBD, whereas atypical symptoms were not noticed by the patient who slept alone in her room. In addition, there was also no evidence of abnormalities in the early PSG assessment. Notably, the abnormal symptoms during sleep worsened with continued use of duloxetine.

Second, the RBD symptoms quickly improved after discontinuing duloxetine for 7 days. In parallel, RSWA sharply decreased then almost completely disappeared after discontinuation of duloxetine for 9 and 37 days, respectively. Previous studies demonstrated that RBD symptoms remained after discontinuing fluoxetine for 6 weeks or even 27 months [14, 15]. Similarly, RSWA persisted for a long time even though the abnormal behaviors had disappeared [16, 17]. In this regard, this case showed rapid improvement in both clinical symptoms and EMG activity during REM sleep.

Lai’s finding confirmed that serotonergic inputs play an important role in redulting spinal motor units in REM sleep [18]. Serotonergic antidepressants, with increasing concentrations of serotonin in the brain, could influence motor tone during REM sleep indirectly at brainstem levels, or directly at spinal levels, and thus produce RSWA [11]. Noradrenaline may also be involved in motor control during REM sleep [19]. As a typical SNRI, the possible mechanism for duloxetine-induced RBD may be related to increasing serotonin and norepinephrine which influence motor tone during REM sleep. Interestingly, Frauscher et al. reported that idiopathic RBD was associated with the increased use of SSRI antidepressants, and was not due to serotonin noradrenaline reuptake inhibitors, or tricyclic antidepressants [20]. Therefore, the exact mechanism of SNRI-induced RBD has not been established. It would be of interest to further explore the potential pathogenesis of RBD secondary to SNRIs in the future.

While our case report indicates that duloxetine can induce RBD, there are three limitations that need to be acknowledged. First, we could not determine the exact time from the point of drug discontinuation to the point when RBD symptoms disappeared. Second, we did not arrange two nights of PSG assessment for the patient at each follow-up, though this may be less of a limitation. The night-to-night variability of RSWA has been widely discussed in previous studies. Cygan et al. found that tonic activity during REM sleep remained stable between nights [21], and Zhang et al. found no difference in phasic and tonic EMG activity between nights [22].Third, we did not conduct tests for neurodegenerative biomarkers, such as the objective smell test and orthostatic systolic blood pressure. As mentioned by Postuma et al. [23], antidepressants are not likely to cause RSWA or RBD, but may unveil RBD at an early point and can thus be a signal of an underlying neurodegenerative disease.

## Abbreviations

AASM: American Academy of Sleep Medicine
AHI: Apnea-hypopnea index
EMG: Chin electromyography
ESS: Epworth Sleep Scale
HAMA: Hamilton Anxiety Scale
HAMD: Hamilton Depression Scale
MRI: Magnetic resonance imaging
PD: Parkinson’s disease
PSG: Polysomnography
RBD: Rapid eye movement behavior disorder
RBDQ-HK: RBD questionnaire
REM: Rapid eye movement
RSWA: REM sleep without atonia
SNRI: Serotonin-norepinephrine reuptake inhibitor
SSRI: Selective serotonin reuptake inhibitor
TCA: Tricyclic antidepressant
TST: Total sleep time
